# Amelioration of Coastal Salt-Affected Soils with Biochar, Acid Modified Biochar and Wood Vinegar: Enhanced Nutrient Availability and Bacterial Community Modulation

**DOI:** 10.3390/ijerph19127282

**Published:** 2022-06-14

**Authors:** Zhangjun Wang, Xin Pan, Shaoping Kuang, Chao Chen, Xiufen Wang, Jie Xu, Xianxin Li, Hui Li, Quanfeng Zhuang, Feng Zhang, Xiao Wang

**Affiliations:** 1 Institute of Oceanographic Instrumentation, Qilu University of Technology (Shandong Academy of Sciences), Qingdao 266001, China; zhangjunwang@qlu.edu.cn (Z.W.); chenchao@qlu.edu.cn (C.C.); wxfsun2005@qlu.edu.cn (X.W.); 10431200541@stu.qlu.edu.cn (J.X.); xianxinli@qlu.edu.cn (X.L.); lihui@qlu.edu.cn (H.L.); zhuangqf@qlu.edu.cn (Q.Z.); 18407303@masu.edu.cn (F.Z.); 2 R & D Center for Marine Instruments and Apparatuses, Pilot National Laboratory for Marine Science and Technology (Qingdao), Qingdao 266200, China; 3 Shandong SCICOM Shenguang Technology Co., Ltd., Qingdao 266300, China; 4 College of Environment and Safety Engineering, Qingdao University of Science and Technology, Qingdao 266042, China; 5 Institute of Coastal Environmental Pollution Control, Key Laboratory of Marine Environment and Ecology, Ministry of Education, Frontiers Science Center for Deep Ocean Multispheres and Earth System, Ocean University of China, Qingdao 266100, China; wangxiao5120@ouc.edu.cn

**Keywords:** soil restoration, biochar, acid-modified biochar, soil microbial communities

## Abstract

As an important part of the ecological environment, degraded coastal soils urgently require efficient and eco-friendly soil amendment. Biochar and wood vinegar have been proved to be effective soil amendments, and acid-modified biochar has great potential in ameliorating the degraded coastal saline–alkali soil. However, the effects of individual or combined application of biochar (BC), acid-modified biochar (ABC), and wood vinegar (WV) on coastal saline–alkali soil are unknown. Hence, biochar, wood vinegar, and acid-modified biochar were prepared by pyrolysis of poplar wood. The properties of biochar were characterized, and soil incubation experiments were conducted. The results showed that ABC decreased the soil alkalinity by acid-base neutralization and improved the soil fertility by increasing the nutrients (C, N, P). ABC provided a more suitable environment and changed the abundance and diversity of soil microorganisms. ABC increased the relative contents of specific families (e.g., *Pseudomonadaceae* and *Sphingomonadaceae*), which had strong ecological linkages in the C, N, and P cycles and organic matter degradation. The results indicated that WV had little effect on coastal saline–alkali soil, whereas individual and combined application of biochar (especially ABC) showed an efficient remediation effect. Our preliminary study demonstrated that the ABC could be a suitable solution for ameliorating degraded coastal saline–alkali soils.

## 1. Introduction

Coastal wetlands are one of the most valuable ecosystems and an important guarantee of ecological security in coastal areas [1]. The Yellow River Delta is a typical coastal estuarine wetland in the world [2]. Owing to economic activities such as oil exploitation, agricultural development, aquaculture, and urbanization, the saline–alkali soil in the Yellow River Delta was seriously polluted, resulting in structural damage or loss of function of coastal saline–alkali soils and aggravating soil salinization [3]. There is an urgent demand to ameliorate coastal saline–alkali soil. Traditional soil remediation measures such as artificial irrigation, humic acid fertilizer, and salt-tolerant plants can alleviate soil salinization to a certain extent, but there are some disadvantages (high cost, low efficiency, and produce secondary pollution) [4]. Therefore, there is an urgent need for efficient and eco-friendly soil amendments to ameliorate the coastal saline–alkali soil.

Biochar is defined as a kind of multi-functional carbon material with abundant functional groups and rich pore structure formed by high-temperature pyrolysis of waste biomass (poultry manure, wood waste, agricultural residue, etc.) under anaerobic or limited oxygen conditions [4]. With its great potential in the field of soil remediation, biochar has gradually become a multi-functional soil amendment [5]. Many studies have shown that biochar can increase the content of soil nutrients and the water holding capacity, which is beneficial to microbial growth [6]. With the abundant pore structure and negatively charged surface functional groups, biochar has high adsorption capacity and high CEC [7], which can alleviate soil salinization [8]. Nevertheless, some studies have suggested the potential negative effect of biochar on soil remediation, such as increasing soil pH and salinity [9] and inhibiting microbial growth [10]. This limits the application of biochar in the restoration of coastal saline–alkali soil. In recent years, modified biochar has gradually become a research hotspot [11]. Compared with the pristine biochar, acid-modified biochar usually have a lower pH [12], contains more O-containing functional groups [13], and the specific surface area and the pore structure are more abundant [14]. This indicated that acid-modified biochar has great potential for ameliorating coastal saline–alkali soil. Whereas the internal relationship between acid-modified biochar and soil nutrients and microorganisms is not clear, and the specific mechanism needs to be further explored.

Wood vinegar is prepared by condensation of biogas produced by anoxic pyrolysis of wood biomass [15]. Wood vinegar is a dark brown liquid with acidic pH [16], and the composition is complex and contains many compounds such as guaiacol, acetic acid, and eugenol [17]. As wood vinegar contains a variety of organic acids, wood vinegar could be used to improve soil pH [18]. In recent years, wood vinegar is often combined with biochar to improve soil properties [19], such as co-applied with biochar to increase blueberry production in farmland soil [20], suppressing the ammonia volatilization from rice paddy soil [21], promoting the growth and development of immature kenaf [22]. However, no attempt has been made to explore the amelioration effects of the independent and combined application of biochar, acid-modified biochar, and wood vinegar on coastal saline–alkali soils, limiting the application of biochar and wood vinegar in soil remediation.

To investigate the independent and combined effect of biochar, acid-modified biochar, and wood vinegar amendment on the coastal saline–alkali soil, a short-term soil incubation trial was carried. The specific objectives of this research were to compare the amelioration effects of the individual and combined application of biochar, acid-modified biochar, and wood vinegar on the salinity, alkalinity, nutrients, and microbial responses of coastal saline–alkali soil. These findings guide the application of biochar and wood vinegar and contribute to the improvement of coastal saline–alkali soil technology.

## 2. Materials and Methods

### 2.1. Soil Sampling

The soil was randomly collected in the Halophytic Botanical Garden of Dongying, located in the Yellow River Delta (118.67° N, 37.42° E), China. The sampling area was planted with okra (*Abelmoschus esculentus* L.) in the past years without fertilizer application before. The soil samples were randomly collected from the topsoil (0–20 cm), air-dried, and ground through a 2 mm sieve.

### 2.2. Preparation of Wood Vinegar and Biochar

Wood vinegar, biochar, and acid-modified biochar were prepared by poplar sawdust in a vacuum tube furnace. The sawdust was charred at 450 °C for 2 h at a heating rate of 10 °C/min under a nitrogen atmosphere (flow rate of 200 mL/min) [23]. Wood vinegar was collected by a cold water circulation system. 180 mL phosphoric acid (H_3_PO_4_) and nitric acid solution (HNO_3_) were fully mixed with 3.6 kg poplar sawdust in a pyrolysis furnace. The preliminary experiment showed that 5% acid addition is the optimal dosage, which can maximize the content of N and P elements in biochar and prevent the structure of biochar from being overoxidized. The solid product prepared under the same pyrolysis conditions was acid-modified biochar (ABC). After the supernatant of WV culture was stored for 3 months under no-light conditions to precipitate the impurities [24], it was filtered with 0.45 μm cellulose acetate membrane and diluted with water at a ratio of 1:500 for further experiment [20]. The biochar was crushed and stored in a bottle through a 50–180-mesh sieve.

### 2.3. Soil Incubation Experiments

200 g air-dried coastal saline–alkali soil samples were put into a ceramic basin and mixed with biochar or wood vinegar. Previous studies have shown that the optimum dosage of biochar is 1.5% (*w*:*w*), and BC, ABC, WV, BC + WV (BWV), and ABC + WV (AWV) are added as the experimental group, and the soil without biochar is used as the control, with three parallel experiments in each group. These treatments were cultured in the greenhouse at 22–26 °C for 45 days. During the experiment, distilled water was added to the soil on time every day, reaching 60% of the field capacity.

### 2.4. Characterization

The functional group of wood vinegar, biochar, and acid-modified biochar was determined by Fourier transform infrared spectrometer (FTIR, Tensor 27, Bruker, Germany). The scanning range was 4000~500 cm^−1^. The composition of wood vinegar was determined by a gas chromatography-mass spectrometer [24]. The initial temperature was 60 °C, 2 min was maintained, and then increased to 280 °C at a 15 °C/min rate to maintain 10 min. The organic C, H, O, and N content of BC and ABC were analyzed by an element analyzer (Vario El Cube, Elementar, Berlin, Germany). The ash content of BC and ABC was determined by the Residual solid mass after heating at 200 °C for 2 h, 400 °C for 2 h, and 600 °C for 1 h. The micropore structure of BC and ABC was tested by scanning electron microscopy (S-480, Hitachi, Tokyo, Japan). The mineral components in BC and ABC were determined by X-ray diffraction (XRD, D8 Advance, Bruker, Germany). The content of the functional groups in BC and ABC were analyzed by X-ray photoelectron spectrometry (Kratos Axis Ultra, Shimadzu, Tokyo, Japan) [25].

### 2.5. Analysis of Soil Property

The pH values of soil samples treated with different treatments were measured by pH meter after collecting water (soil-water ratio 1:1:2.5). Soil organic carbon was determined by the high-temperature external heating method of potassium dichromate and concentrated sulfuric acid [23]. Soil total nitrogen and active nitrogen were determined by an automatic discontinuous chemical analyzer (KDN-102F; Qianjian Ltd., Shanghai, China). Soil total phosphorus and total potassium were determined by an inductively coupled plasma multi-channel direct reading spectrometer (Nexion 350X; PerkinElmer, Waltham, MA, USA). Soil available phosphorus was determined by spectrophotometer (UV-8000 T, Shanghai Meitash Instrument Co., Ltd., Shanghai, China). According to the manufacturer’s instructions, the soil microbial genomic DNA was extracted from approximately 1 g (wet weight) of soil using a Mobio PowerSoil DNA separation kit (Mobio Labs, Carlsbad, CA, USA). After DNA extraction from soil samples, 1% agarose gel electrophoresis was used to detect the extracted genomic DNA. The amplified products were detected with 2% agarose gel. The sample is mixed into the same amount according to the concentration of the product of the polymerase chain reaction. After fully mixing, the gel recovery kit provided by the Qiagen Company was used to recover the target strip products. Finally, the library was constructed with TruSeq ^®^DNA non-polymerase chain reaction kit. The constructed library was quantitatively detected by Qubit and Q-PCR. After the library was qualified, HiSeq2500 PE250 was used to sequence it on the computer.

### 2.6. Statistical Analysis

To evaluate the significance of the soil properties and the effects of BC, ABC, and WV amendments on the soil physicochemical properties, an Analysis of variance (ANOVA) followed by Duncan multiple comparison tests was performed using SPSS25.0 software (IBM Corp., New York, NY, USA).

## 3. Results and Discussion

### 3.1. Characterization of Wood Vinegar and Biochar

**Figure 1:**
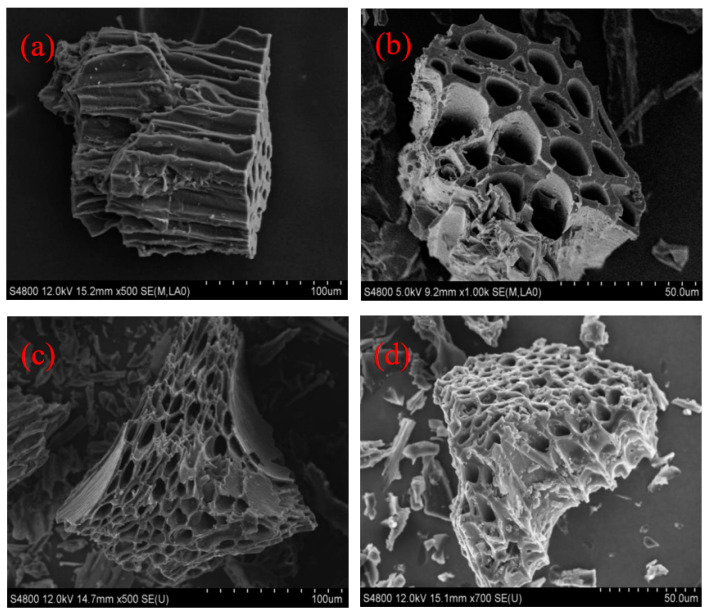
SEM images of the sawdust-derived biochar (BC) (**a**,**b**) and acid-modified biochar (ABC) (**c**,**d**).

**Figure 2:**
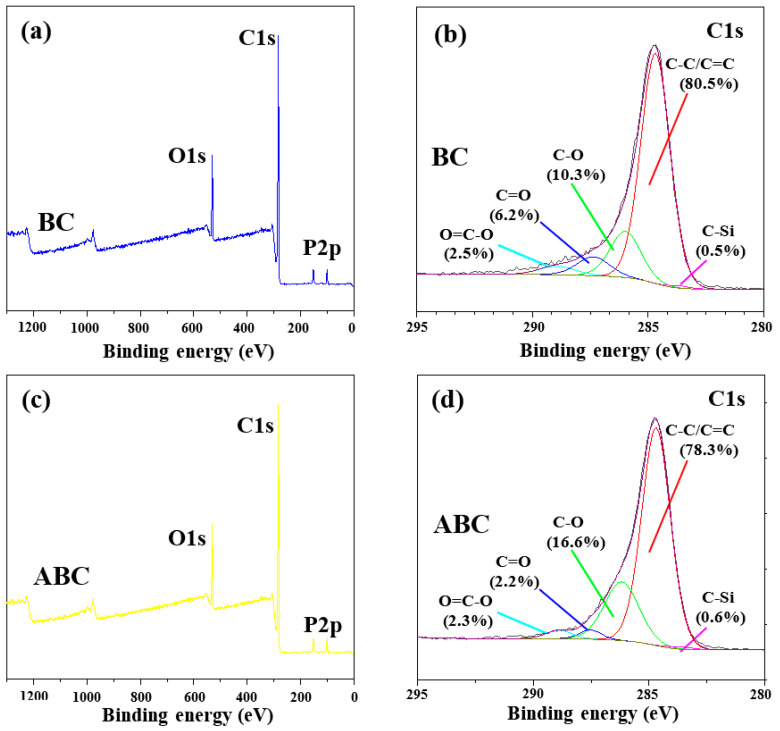
The full spectra (**a**,**c**) and high-resolution XPS spectra of C1s (**b**,**d**) of the biochar and acid-modified biochar.

As shown in the picture, the surface of sawdust biochar is relatively smooth and shows a regular porous block structure. Sawdust biochar basically maintains the original shape of sawdust, and its pore structure is abundant and orderly (Figure 1a,b). After being modified by acid, the pores of ABC are more developed (Figure 1c,d). Similar results were acquired from previous reports [26]. The results showed that phosphoric acid and nitric acid, as solid, strong acids, can corrode the surface structure of BC and make its surface rough. XPS spectra in Figure 2 further showed the surface elemental compositions and valence states of C and P on the biochar surface. Clearly, the peak at 284.8 ev, 286.5 ev, 287.8 ev, and 288.7 ev represents the C=C/C–C/C–H, C–O, C=O, and O=C–O functional groups [27], respectively. Compared to BC, the relative contents of C–O functional groups on the ABC surface slightly enhanced from 10.3% to 16.3% and the contents of C=O functional groups weakened from 6.2% to 2.2% (Figure 2b,d), respectively. It indicated that HNO_3_ and H_3_PO_4_ modified the composition of oxygen-containing functional groups of biochar.

**Figure 3:**
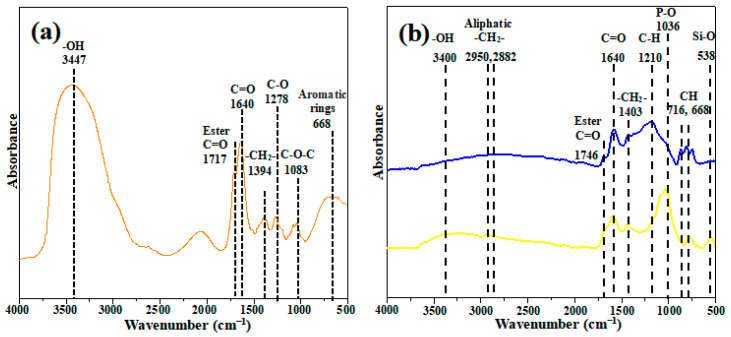
The FTIR spectra of the wood vinegar (WV) (**a**) and biochar (BC, ABC) (**b**).

Figure 3 showed the FTIR spectrum of BC, ABC and WV, the absorption peak at 3500–3400 cm^−1^ represents the vibration of O-H 2950 and 2882 cm^−1^ correspond to the asymmetric and symmetrical stretching vibration of alkyl CH_2_, respectively. The absorption peaks at wavenumber 1740 and 1640 cm^−1^ represent the C=O bond in lipids and ketones, respectively, while the C bond mainly appears at 1521 cm^−1^ and 1440 cm^−1^, which mainly represents the asymmetric bending vibration of -CH_2_-. The Omure H bond of the phenolic hydroxyl group mainly appears at 1385 cm^−1^, while the C–O bond at 1335 cm^−1^ represents the C–O bond in the carboxyl group, while the peak of C–O–C and Pmure O bond appears at 1050 and 1041 cm^−1^, while the peak at 600 cm^−1^ represents the Si-O bond [28]. ABC contains Pmuro bond at wavenumber 1041 cm^−1^. This also further indicates the phosphorus oxides, phosphoric acid, and (hydro)phosphates appeared on the biochar.

Both BC and ABC have two distinct characteristic peaks at the sum of 2θ, corresponding to the microcrystalline structure of cellulose and hemicellulose [29], respectively (Figure S1). In comparison, the characteristic peak of calcium pyrophosphate (Ca_2_P_2_O_7_) in ABC is more obvious.

Furthermore, (O+N)/C and H/C atomic ratios are commonly applied to characterize the polarity and aromaticity of biochar. In this paper, the (O+N)/C and O/C atomic ratios of ABC were both larger than those of BC, demonstrating that the modification of phosphoric acid and nitric acid could increase the polarity and decline the aromaticity of the biochar [30].

A total of 59 compounds in wood vinegar were detected by GC-MS, indicating that wood vinegar contains a lot of phenols and organic acids. Organic functional groups in wood vinegar, such as C=O bonds of 1746 cm^−1^ and 1640 cm^−1^, were identified by FTIR, which indicated carboxyl and esters, revealing that the main components of PWV were carboxylic acids, esters, aldehydes, or ketones. Table S1 shows that this is consistent with the results of GC-MS [31].

### 3.2. Improvement of Soil Properties and Nutrients

**Figure 4:**
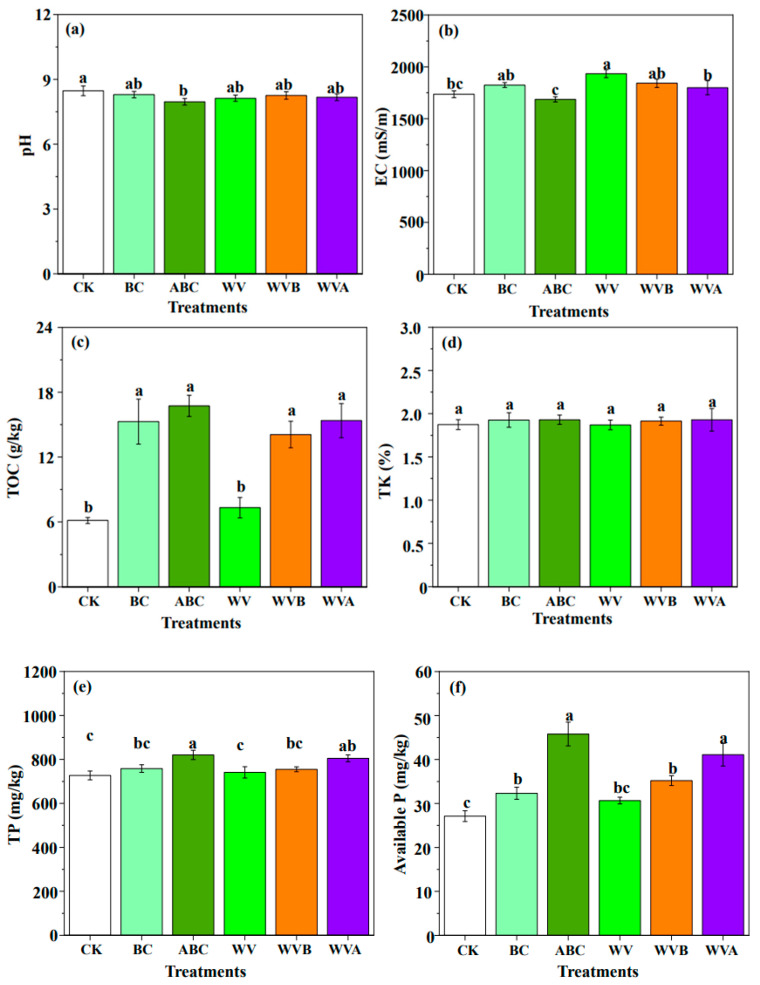

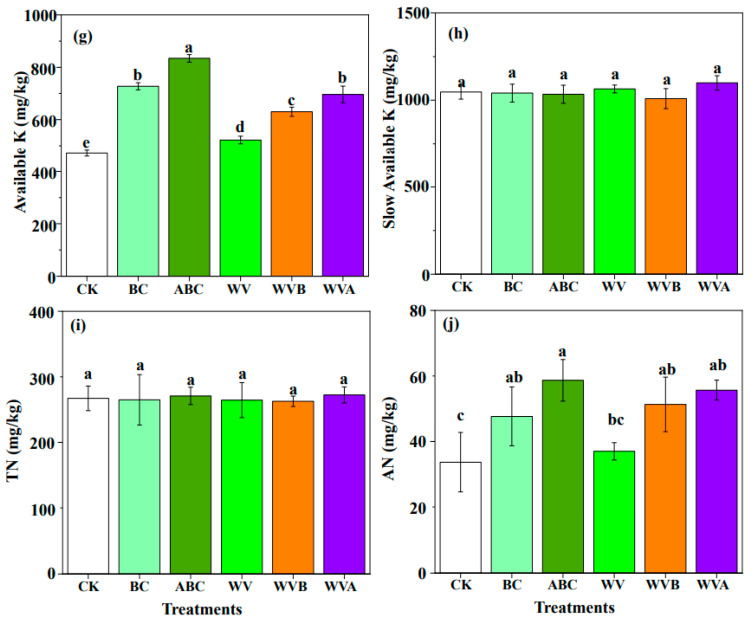
The transformations in pH (**a**), Electrical conductivity (**b**), Total organic carbon (**c**), Total potassium (**d**), Total phosphorus (**e**), Available phosphorus (**f**), Available potassium (**g**), Slow available potassium (**h**), Total nitrogen (**i**), and Available nitrogen (**j**) in different treatments (CK, BC, ABC, WV, WVB, and WVA) of coastal saline–alkali soil. The different letters among different treatments indicate significant differences, which were analyzed by Duncan’s test (*p* = 0.05).

The effects of individual and co-application of BC, ABC, and WV on soil properties are shown in Figure 4. Remarkably, ABC significantly decreased the soil pH to 8.26 due to the acid-base neutralization reactions between the nitric acid and phosphoric acid in ABC and the hydroxyl ion in soil [32]. This consisted of the results in Table 1. This effect may affect the availability of nutrient elements, soil microbial community structure, and organic matter content [33]. In addition, the pH of WV is 4.60, the addition of WV may lead to a reduction in soil pH, but with 45 days of culture, the organic acids in WV were consumed [24], resulting in no obvious changes in the pH of WV, WVB, and WVA. The EC values of BC and ABC showed non-significant differences with CK; this was consistent with a previous study [14].

Figure 4c shows that all treatments significantly increased soil total organic carbon concentrations, especially ABC; this is attributed to the fact that biochar contains a large amount of aromatic carbon, while the organic matter in WV is degraded in soil, resulting in less TOC in WV than other treatment groups. As shown in Figure 4d, the concentration of TK is not significantly different in each treatment, which could be attributed to the fact that there was no K element in the feedstock. The available K in BC, ABC, WVB, and WVA significantly improved than CK. This opposite trend of increasing K^+^ concentration in soils that are quickly absorbed by plants may be attributed to biochar surfaces containing a large number of negatively charged oxygen-containing functional groups, which could also be found in other studies [34]. In addition, the adsorption of nutrient salts by biochar could also explain the alleviating effect of ABC and BC on soil salinization [35].

Figure 4e,f shows that all treatments significantly increased soil total P and available P concentrations, especially for the ABC and WVA treatments, which led to the greater available P concentrations. This may be attributed to the greater contents of P in ABC after being modified by H_3_PO_4_. This finding also suggests that the P loaded on biochar could serve as an important supplement for soil P. The results were consistent with previous findings that biochar contained a certain amount of P and could enhance the soil total and available P; similar shifts occur in soil nitrogen (Figure 4i,j).

Furthermore, at the same level of addition, the available N of soils treated with ABC was significantly greater than the CK soil and BC-treated soil. Previous literature has also shown that the availability of soil nutrient elements is highly related to pH because pH can affect the type and intensity of metal ions (such as calcium, potassium, and magnesium) in soil [36], which may be another reason why ABC plays a significant role in increasing soil total phosphorus and available phosphorus. Therefore, ABC can be used as a kind of fertilizer, which can store and release soil nutrients (i.e., N, P), which is beneficial to the reproduction of microorganisms.

### 3.3. Changes in Soil Microbial Diversity

**Figure 5:**
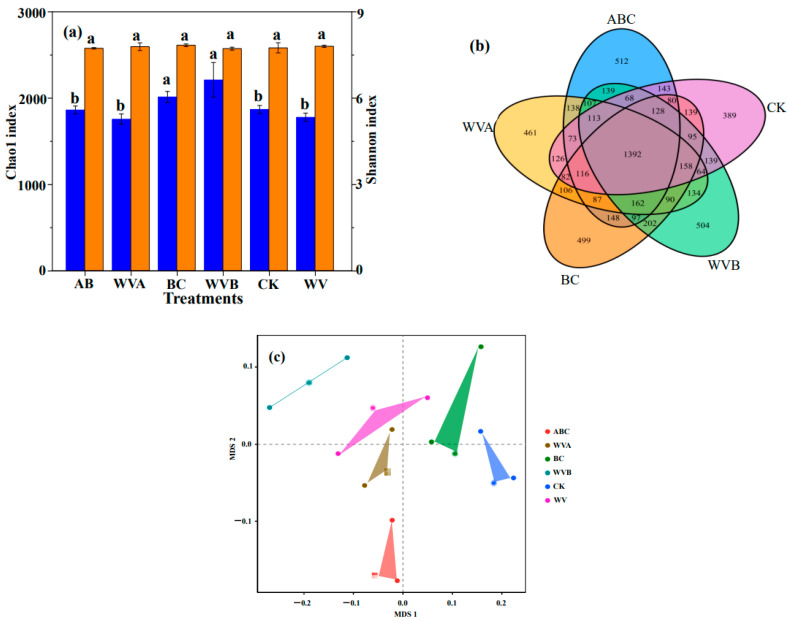
Profiles of the bacterial community in the coastal saline–alkali soil with different treatments. (**a**) The richness (Chao1) and diversity (Shannon) index for the soil bacterial community. (**b**) Venn diagram showed the shared bacterial OTUs. (**c**) Non-metric multidimensional scaling (NMDS) ordination plots of bacterial community composition based on Bray–Curtis similarity. The different letters among different treatments indicate significant differences, which were analyzed by Duncan’s test (*p* = 0.05).

**Figure 6:**
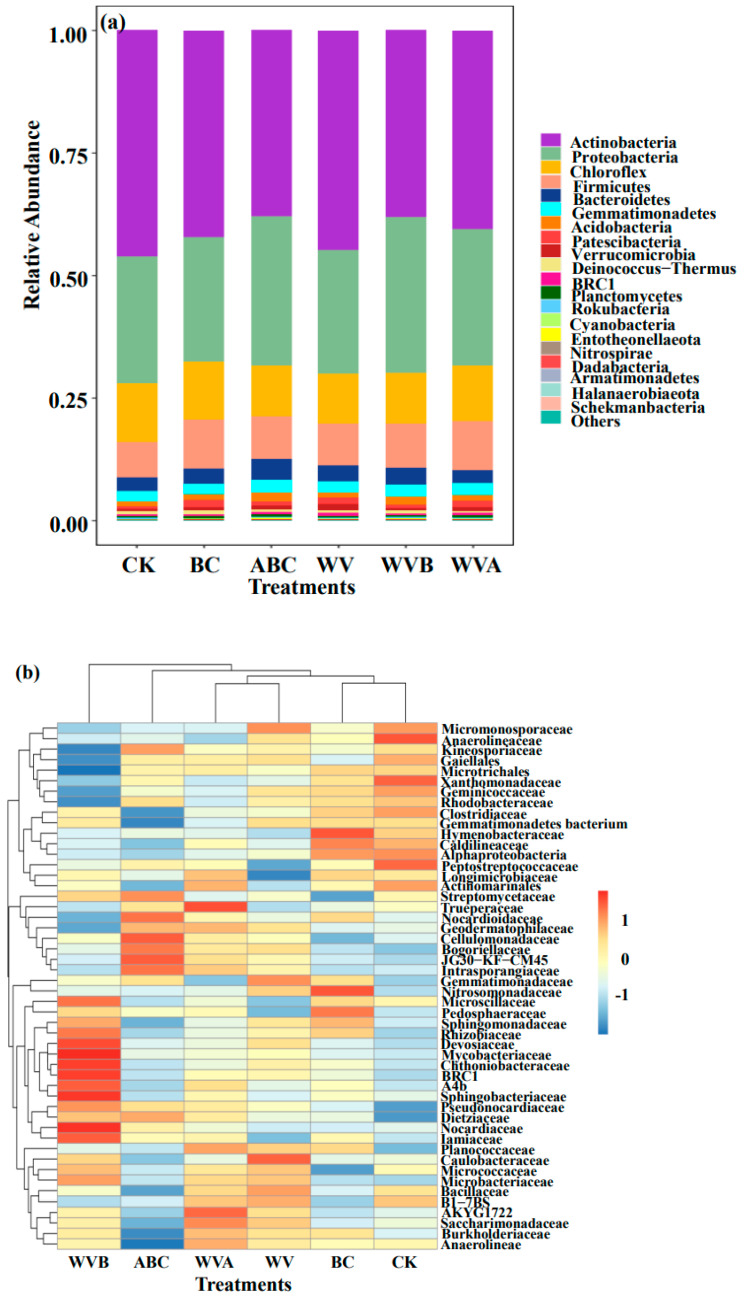

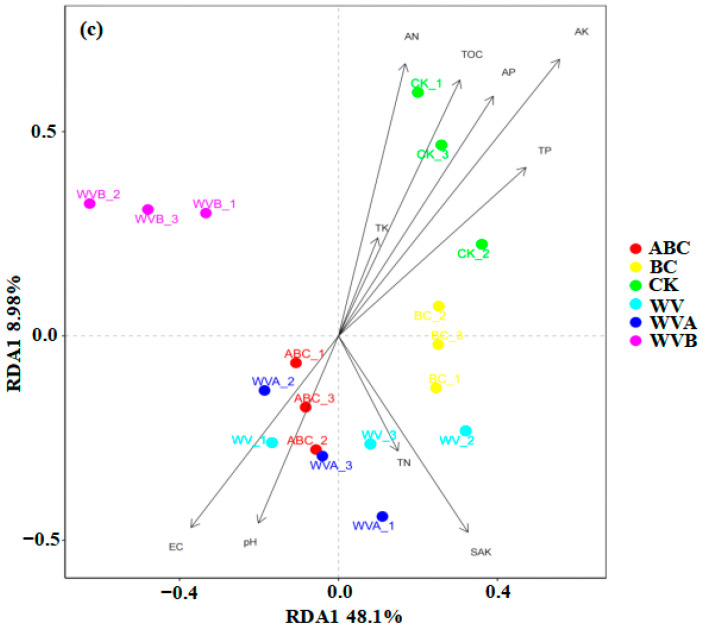
(**a**) The relative abundance of the top 10 bacterial phyla in the coastal saline–alkali soil, the heat map of the top 35 bacterial at the family level. (**b**) The color intensity of the scale demonstrates the relative abundance of each genus. (**c**) Redundancy analysis (RDA) between microbial family relative abundance and soil properties in different treatments.

As an important part of the soil environment, soil microorganism is an important index that reflects the properties of soil. Applying different soil amendments has different effects on the soil microbial community. As shown in Figure 5a, the individual addition of BC and ABC or the combined addition with WV will increase the richness of the bacterial community of saline–alkali soil, whereas the application of WV had a weak effect on bacterial abundance and all treatments had little effect on the diversity of soil bacteria. These results demonstrated that a dramatic change occurred in the soil microbial diversity. The previous study has shown that biochar could promote the proliferation of microorganisms by increasing soil organic carbon, nitrogen, and phosphorus [37]. This was consistent with the results in 3.2. The Venn diagram showed that the individual or combined application of BC and ABC with WV had an effect on the microbial community (Figure 5b), the total number of OTU in the four treatment groups and CK was 1392, while the unique OTU number of CK, BC, ABC, WVB, and WVA was 389, 499, 512, 504, and 461, respectively, and there were significant changes in bacterial diversity among different treatments. This result further indicated that the application of BC and ABC changed the soil bacterial community composition. A similar result was observed by Zoug et al. [38], who found that the application of biochar could significantly increase the number of unique OTUs, indicating that there were more unique bacterial species with biochar treatment. This conclusion is confirmed by the obvious aggregation of the soil bacterial community (Figure 5c). The phylum-level microbial community structure of CK and five treatments were shown in Figure 6a, *Actinobacteria*, *Proteobacteria*, *Chloroflexi*, *Firmicutes*, *Bacteroidetes*, *Gemmatimonadetes*, *Acidobacteria*, and *Patescibacteria* is the top 8 species of phylum microorganisms with the highest abundance in soil, which accounted for 96.2–97.7%. Compared with CK, the relative abundance of *Proteobacteria* was increased by adding each treatment group, and the abundance of *Proteobacteria* was positively correlated with the content of nutrient elements in the soil. The response of the microbial community to the addition of biochar was mainly attributed to the fact that BC and ABC increased the active nutrient elements in the soil and modified the substrate (e.g., C, N, and P) effectiveness.

Similar results were found in a previous study; the application of biochar could increase the relative contents of *Acidobacteria*, while *Proteobacteria* could benefit soil C storage via producing microbial mucilages and polysaccharides in favor of stabilizing soil aggregates [22]. In addition, the addition of BC (especially ABC) significantly increased the abundance of Acidobacteria, indicating that the change in soil pH was more suitable for the growth of Acidobacteria, which was consistent with the previous data [39]. At the family level, the application of ABC and WV has increased the relative contents of specific families (such as *Pseudomonadaceae* and *Sphingomonadaceae*). According to previous research [37], these families had strong ecological linkages in the C, N, and P cycles and organic matter degradation. RDA revealed the relationship between soil properties and microbial community structure in different treatments. Figure 6 revealed that the change of microbial community is positively correlated with the content of nutrient elements in soil (especially AK, AP, AN, and TOC) and negatively correlated with pH and electrical conductivity. Previous studies have shown that biochar can provide nutrients for microorganisms and promote soil circulation [40]. Especially ABC can provide more N and P than BC and WV. In addition, ABC with a more developed pore structure can provide more surface functional groups, and ABC is the most effective treatment in alleviating salt pressures in saline–alkali soil, which positively impacts microbial diversity and activity.

## 4. Conclusions

To the best knowledge, this is the first study to investigate the amelioration effects of the individual and combined application with biochar, acid-modified biochar, and wood vinegar on coastal saline–alkali soils. Owing to the rapid consumption of active substances in WV, WV has little effect on coastal saline–alkali soil. The application of BC (especially the application of ABC) has been proved to improve soil pH, alleviate salt stress through adsorption and increase nutrients in the soil. In addition, the individual and combined application with BC, ABC, and WV could increase the abundance of specific microorganisms (e.g., *Pseudomonadaceae* and *Sphingomonadaceae*) and promote the shift of bacterial communities to bacterial functional taxa (e.g., *Proteobacteria* and *Acidobacteria*). Compared with BC, ABC could not only expand the pore structure but also contain more oxygen-containing functional groups. Benefited from the special properties, ABC had the best effect in alleviating the adverse effects of environmental soil salt ion stress and neutralizing soil pH. The results showed that ABC could be an efficient saline–alkali soil amendment. However, the combined application of biochar, acid-modified biochar, and wood vinegar had no obvious effect on soil properties, and the related mechanism study needs to be further explored. This work can provide guidance for the practical application of biomass pyrolysis products (biochar and wood vinegar) in the field of coastal saline–alkali soil remediation.

## Figures and Tables

**Table 1:** Selected characteristics of the sawdust biochar, acid-modified biochar, and wood vinegar.

Samples	Yield %	Elemental Composition (wt%)				Atomic Ratio			pH	Ash%
		C%	H%	O%	N%	H/C ^a^	O/C ^a^	(O + N)/C ^a^		
BC	36.5	77.3	5.64	15.3	1.76	0.87	0.15	0.17	8.53 ± 0.06	1.35
ABC	33.8	74.1	4.51	19.5	1.89	0.73	0.20	0.22	8.14 ± 0.04	1.10
WV	31.7	nd	nd	nd	nd	nd	nd	nd	4.60 ± 0.0	nd

^a^ H/C: atomic ratio of hydrogen to carbon. O/C: atomic ratio of oxygen to carbon. (N + O)/C: atomic ratio of the sum of nitrogen and oxygen to carbon.

## Data Availability

Data are not publicly available, though the data may be made available on request from the corresponding author.

## Supplementary Materials

The following supporting information can be downloaded at: https://www.mdpi.com/article/10.3390/ijerph19127282/s1, Figure S1: XRD spectra of the biochar (BC) and acid modified biochar (ABC); Table S1: The main components of the poplar derived wood vinegar (WV) analyzed by GC-MS.
