# Supplementary material for: Amelioration of Coastal Salt-Affected Soils with Biochar, Acid Modified Biochar and Wood Vinegar: Enhanced Nutrient Availability and Bacterial Community Modulation
Source: Int J Environ Res Public Health. 2022 Jun 14;19(12):7282. doi: 10.3390/ijerph19127282 (PMC9223450; doi:10.3390/ijerph19127282)
Supplement: Supplementary file 1 [file ijerph-19-07282-s001.zip › ijerph-1711890-supplementary.pdf]

Supplementary Information

Amelioration of Coastal Salt-Affected Soils with Biochar, Acid Modified Biochar and Wood Vinegar: Enhanced Nutrient Availability and Bacterial Community Modulation

Zhangjun Wang [1,2,3], Xin Pan [1,*], Shaoping Kuang [4,*], Chao Chen [1,2,3], Xiufen Wang [1,3], Jie Xu [1], Xianxin Li [1,2], Hui Li [1], Quanfeng Zhuang [1], Feng Zhang [1] and Xiao Wang [5]

[1] Institute of Oceanographic Instrumentation, Qilu University of Technology (Shandong Academy of Sciences), Qingdao 266001, China; zhangjunwang@qlu.edu.cn (Z.W.); chenchao@qlu.edu.cn (C.C.); wxfsun2005@qlu.edu.cn (X.W.); 10431200541@stu.qlu.edu.cn (J.X.); xianxinli@qlu.edu.cn (X.L.); lihui@qlu.edu.cn (H.L.); zhuangqf@qlu.edu.cn (Q.Z.); 18407303@masu.edu.cn (F.Z.)

[2] R & D Center for Marine Instruments and Apparatuses, Pilot National Laboratory for Marine Science and Technology (Qingdao), Qingdao 266200, China

[3] Shandong SCICOM Shenguang Technology Co., Ltd., Qingdao 266300, China

[4] College of Environment and Safety Engineering, Qingdao University of Science and Technology, Qingdao 266042, China

[5] Institute of Coastal Environmental Pollution Control, Key Laboratory of Marine Environment and Ecology, Ministry of Education, Frontiers Science Center for Deep Ocean Multispheres and Earth System, Ocean University of China, Qingdao 266100, China; wangxiao5120@ouc.edu.cn

* Correspondence: panxin@poers.edu.pl (X.P.); spkuang@qust.edu.cn (S.K.); Tel.: +86-532-58628657 (X.P.); +86-532-88959332 (S.K.)

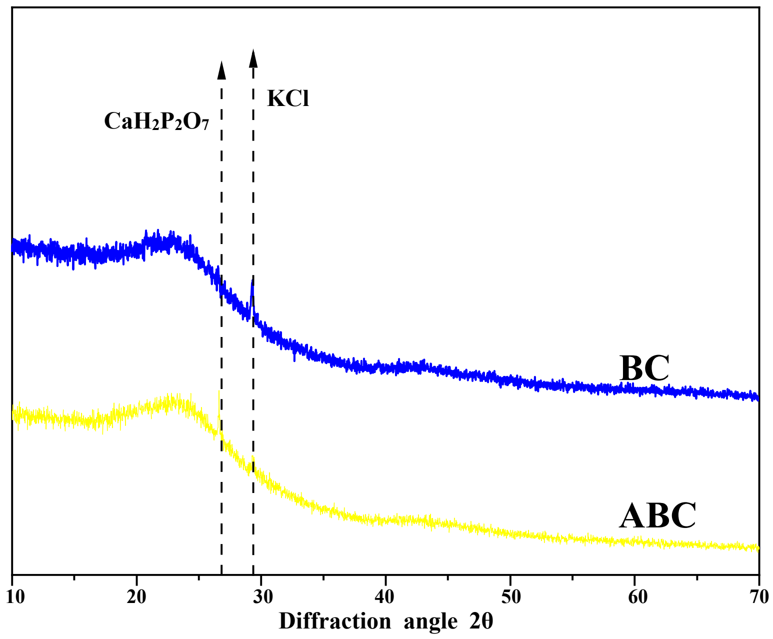

Figure S1. XRD spectra of the biochar (BC) and acid modified biochar (ABC).

Table S1. The main components of the poplar derived wood vinegar (WV) analyzed by GC-MS.

No.	RT(min)	Compounds	Molecular Formula	CAS Number	RA (%)
1	4.8	2-Butanone, 3-hydroxy-	$C_4H_8O_2$	000513-86-0	1.05
2	5.383	2-Cyclopenten-1-one, 2-methyl-	C_6H_8O	001120-73-6	0.17
3	5.775	2-Cyclopentenone	C_5H_6O	000930-30-3	2.82
4	5.923	2-Cyclopenten-1-one, 2-methyl-	C_6H_8O	001120-73-6	1.80
5	6.198	Acetic acid	$C_2H_4O_2$	000064-19-7	28.6
6	6.95	Furfural	$C_5H_4O_2$	000098-01-1	1.95
7	7.247	Hydrazine, methyl-	CH_6N_2	000060-34-4	0.48
8	7.586	Propanoic acid	$C_3H_6O_2$	000079-09-4	3.51
9	7.808	2-Cyclopenten-1-one, 3-methyl-	C_6H_8O	002758-18-1	1.95
10	7.988	Propanoic acid, 2-methyl-	$C_4H_8O_2$	000079-31-2	0.42
11	8.036	2-Cyclopenten-1-one, 2,3-dimethyl-	$C_7H_{10}O$	001121-05-7	0.81
12	8.205	Pentanoic acid, 4-oxo-, methyl ester	$C_6H_{10}O_3$	000624-45-3	0.17
13	8.348	2-Furancarboxaldehyde, 5-methyl-	$C_6H_6O_2$	000620-02-0	2.02
14	8.666	Butanoic acid	$C_4H_8O_2$	000107-92-6	2.90
15	8.777	2-Propenoic acid	$C_3H_4O_2$	000079-10-7	0.26
16	8.952	Butyrolactone	$C_4H_6O_2$	000096-48-0	1.79
17	9.084	2-Cyclopenten-1-one, 3-ethyl-	$C_7H_{10}O$	005682-69-9	0.22
18	9.476	2(5H)-Furanone, 5-methyl-	$C_5H_6O_2$	000591-11-7	0.64
19	9.921	2(5H)-Furanone, 3-methyl-	$C_5H_6O_2$	022122-36-7	1.16
20	10.297	2(5H)-Furanone	$C_4H_4O_2$	000497-23-4	0.87
21	10.329	Crotonic acid	$C_4H_6O_2$	003724-65-0	0.97
22	10.408	Mepivacaine	$C_{15}H_{22}N_2O$	000096-88-8	0.87
23	10.646	2-Cyclopenten-1-one,	$C_7H_{10}O_2$	021835-00-7	0.70
24	10.848	2-Cyclohexen-1-ol	$C_6H_{10}O$	000822-67-3	0.14
25	11.054	2-Cyclopenten-1-one, 2-hydroxy-3-methyl-	$C_6H_8O_2$	000080-71-7	4.82
26	11.303	Phenol, 2-methoxy-	$C_7H_8O_2$	000090-05-1	4.82
27	11.61	2,3-Dimethyl-4-hydroxy-2-butenoic lactone	$C_6H_8O_2$	001575-46-8	0.2`
28	11.689	2-Cyclopenten-1-one, 3-ethyl-2-hydroxy-	$C_7H_{10}O_2$	021835-01-8	0.73
29	11.78	4-Methyl-5H-furan-2-one	$C_5H_6O_2$	006124-79-4	0.98
30	12.166	2-Methoxy-5-methylphenol	$C_8H_{10}O_2$	001195-09-1	0.17
31	12.272	Phenol, 2-methoxy-4-methyl-	$C_8H_{10}O_2$	000093-51-6	3.73
32	12.489	Maltol	$C_6H_6O_3$	000118-71-8	1.46
33	12.674	Phosphonic acid, (p-hydroxyphenyl)-	$C_6H_7O_4P$	033795-18-5	2.57
34	12.955	Phenol, 4-ethyl-2-methoxy-	$C_9H_{12}O_2$	002785-89-9	1.25
35	13.495	Phenol, 4-methyl-	C_7H_8O	000106-44-5	1.19
36	13.681	Phenol, 2-methoxy-4-propyl-	$C_{10}H_{14}O_2$	002785-87-7	0.51
37	14.109	1,4:3,6-Dianhydro-.alpha.-d-glucopyranose	C_6H_8O4	1000098-14-8	0.79
38	14.215	Butanoic acid, anhydride	$C_8H_{14}O_3$	000106-31-0	1.55
39	14.305	Phenol, 3-ethyl-	$C_8H_{10}O$	000620-17-7	0.27
40	14.718	Phenol, 3,4-dimethyl-	$C_8H_{10}O$	000095-65-8	0.19
41	15.56	Pentanoic acid, 4-oxo-	$C_5H_8O_3$	000123-76-2	1.12
42	15.867	Phenol, 2-methoxy-4-(1-propenyl)-, (Z)-	$C_{10}H_{12}O_2$	005912-86-7	0.40
43	16.069	2,3-Anhydro-d-mannosan	$C_6H_8O_4$	1000129-98-0	0.62
44	16.397	Benzoic acid	$C_7H_6O_2$	000065-85-0	0.37
45	16.508	3-Pyridinol	C_5H_5NO	000109-00-2	0.16
46	17.133	2-Furancarboxaldehyde, 5-(hydroxymethyl)-	$C_6H_6O_3$	000067-47-0	1.27
47	17.668	Vanillin	$C_8H_8O_3$	000121-33-5	0.94
48	17.811	Isosorbide	$C_6H_{10}O_4$	000652-67-5	0.20
49	18.224	Ethanone, 1-(4-hydroxy-3-methoxyphenyl)-	$C_9H_{10}O_3$	000498-02-2	0.94
50	18.494	1,2-Benzenediol	$C_6H_6O_2$	000120-80-9	4.19
51	19.034	1,2-Benzenediol, 4-methyl-	$C_7H_8O_2$	000452-86-8	2.89
52	19.712	4-Ethylcatechol	$C_7H_8O_2$	001124-39-6	0.79
53	20.247	2,5-Dimethylhydroquinone	$C_8H_{10}O_2$	001321-28-4	0.07
54	20.543	1,4-Benzenediol, 2,6-dimethyl-	$C_8H_{10}O_2$	000654-42-2	0.15
55	20.586	Benzeneacetic acid, 4-hydroxy-3-methoxy-	$C_9H_{10}O_4$	000306-08-1	1.14
56	20.771	1,4-Benzenediol, 2-methyl-	$C_7H_8O_2$	000095-71-6	1.04
57	20.967	Hydroquinone	$C_6H_6O_2$	000123-31-9	1.23
58	21.83	9,12-Octadecadienoic acid (Z,Z)-	$C_{18}H_{32}O_2$	000060-33-3	0.16
59	23.18	D-Allose	$C_6H_{12}O_6$	002595-97-3	0.82
